# Understanding Cannabinoid Psychoactivity with Mouse Genetic Models

**DOI:** 10.1371/journal.pbio.0050280

**Published:** 2007-10-09

**Authors:** Ken Mackie

## Abstract

Thanks to recent advances in the cannabinoid field, investigators have begun to relate cannabis's cellular mechanisms to its psychoactive behavioral effects.

The fact that cannabis is the most widely used illicit drug has motivated a great deal of research aimed at understanding how it produces its psychoactive effects. Here I use the term psychoactive to describe the mild euphoria, altered perceptions, sense of relaxation, and sociability that often, but not always, accompany recreational cannabis use. Despite the difficulties inherent in working with lipophilic cannabinoids such as Δ^9^-tetrahydrocannabinol (THC), which is the primary psychoactive component of cannabis, our understanding of the mechanism of action of these compounds at the cellular level has increased dramatically over the past 20 years. However, a complete understanding of how cannabis elicits its psychoactive effects would include an appreciation of its actions at the cellular and network level as well as an identification of the neural circuits perturbed. The cannabinoid field has now matured to the point where investigators can begin to relate the cellular mechanisms of THC action to the behavioral effects of cannabis.

Research during the past 20 years has led to the discovery of the endocannabinoid system, or ECS. The ECS is composed of cannabinoid receptors, endogenous cannabinoids (endocannabinoids), and the enzymes that synthesize and degrade the endocannabinoids [1,2]. There are two well-characterized cannabinoid receptors, CB1 and CB2, both of which are G protein-coupled receptors (GPCRs) [3]. I will focus on CB1, which appears to mediate most of the psychoactive effects of cannabis. Binding, in situ hybridization, and immunocytochemical studies reveal a striking pattern of CB1 expression. The receptor is one of the most abundantly expressed GPCRs in the brain and is particularly enriched in the cortex, amygdala, hippocampus, basal ganglia, and cerebellum [4,5]. CB1 receptors are highly expressed on axons and axon terminals, where they are well positioned to modulate neurotransmission [6]. Indeed, endocannabinoids produced by neurons or glia mediate several forms of transient and persistent synaptic plasticity [7]. In addition to these synaptic actions, the activation of somatic CB1 receptors inhibits neuronal excitability in some forebrain and cerebellar neurons [8,9]. Given the widespread role of CB1 receptors and endocannabinoids in eliciting or shaping neuronal plasticity, it is reasonable to speculate that THC and other cannabinoids produce their psychoactive effects by perturbing endocannabinoid-mediated plasticity or neuronal excitability.

A fundamental issue when studying the mechanism of action of psychoactive drugs is choosing an animal model that best represents the effects of the drug in humans. When knockout mice are used, this question becomes choosing the appropriate mouse model. Of course, before a model is chosen, the particular effect(s) being modeled need to be determined. In the case of commonly abused drugs, these effects may range from therapeutic (e.g., analgesia) to subjective (e.g., relaxation, altered perception, etc.) to reinforcing (e.g., self-administration, craving, etc.) actions of the drug. Reliable models for some of these behaviors—e.g., analgesia, self-administration, relapse, sensitization, etc.—have been developed, but models for more subjective effects such as hallucinations and subtle alterations in perception have been more difficult to establish. Indeed, one might argue that rodents won't experience some of these subjective effects, so efforts in this direction may be futile. Also complicating model design and choice is that many drugs of abuse, including cannabis, elicit biphasic effects in humans where at a low dose (or in some subjects), positive effects dominate, but at higher doses, the opposite is observed. For example, low doses of THC are rewarding, while higher doses are aversive, and still higher doses cause motor impairment that obscures any rewarding or aversive effects [10]. Thus, it is important that full dose-response studies are conducted in the chosen animal model.

Animal models for the behavioral effects of cannabinoids have primarily focused on prediction of cannabis-like activity, drug discrimination, and memory disruption. The dog static ataxia test is historically significant. Studies using this model were among the first to strongly suggest that THC produced behavioral effects via a receptor, rather than by nonspecific membrane interactions. The administration of cannabinoid compounds to a dog causes the dog to weave back and forth while remaining in one place; the term “static ataxia” was coined to describe this peculiar collection of behaviors. Compounds that produced static ataxia in the dog had a high likelihood of eliciting cannabis-like psychoactivity in humans, and it was possible to develop a structure-activity relationship for cannabinoids using this test [11]. These findings, plus the observation that static ataxia is blocked by the CB1 receptor antagonist SR141716 (rimonabant), strongly suggest that both are mediated by CB1 receptors [12]. However, as with most animal behavioral responses to cannabinoids, these observations do not necessarily imply (indeed it is highly unlikely) that the neural circuits underlying static ataxia are the same as those producing psychoactivity in humans.

The dog static ataxia test appears accurate in predicting cannabis-like psychoactivity of a compound; however, it and other tests such as drug discrimination and memory tasks are either financially costly or require time-consuming subject training to perform, which are drawbacks limiting their widespread use. The shortcomings of the available models for screening cannabinoid-like compounds prompted a continuing search for alternatives and led to the development of the mouse “tetrad” in the mid-1980s by Billy Martin's group [13]. The tetrad consists of four individual tests: analgesia (typically by tail withdrawal or flick), sedation, catalepsy, and hypothermia. Although none of these tests individually is predictive of cannabis-like activity, the presence of the four responses often, but not invariably [14], correlates with a drug having either cannabis-like psychoactivity and/or agonist activity at CB1 receptors. A distinct advantage of the tetrad is that its components can be measured sequentially after drug administration, greatly simplifying behavioral assay design. Because of its robustness and relative ease of use, the tetrad has assumed a place of central importance in behavioral assays of cannabinoid action.

A large number of studies demonstrate that THC or potent synthetic cannabinoids produce the tetrad by activating CB1 receptors. The most compelling of these include antagonism of the tetrad by CB1 receptor antagonists such as rimonabant [15] and its absence in CB1 receptor knockout mice [16]. Despite the tetrad's importance for studying the behavioral pharmacology of cannabinoids, little is known about which neurons underlie its components. It is tempting to speculate that the neurons expressing the highest levels of cannabinoid receptors might be important for producing the tetrad. In the cortex, amygdala, and hippocampus, CB1 is highly expressed in a subclass of large-diameter, cholecystokinin (CCK)-positive GABAergic interneurons (those that release aminobutyric acid), for example, [17]. However, CB1 receptors are also extensively expressed at lower levels in a wide variety of neurons, including glutamatergic principal cells throughout the forebrain [18–20]. This is illustrated for the hippocampal formation in Figure 1. Thus, understanding how THC produces the behaviors measured in the tetrad depends on determining which neurons are involved.

**Figure 1:**
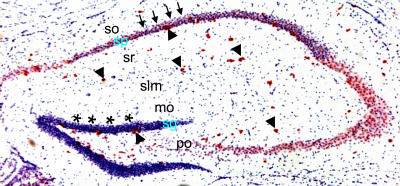
CB1 Expression Levels Vary Widely in the Mouse Hippocampal Formation. In cortex, CB1 mRNA is highly expressed in a very restricted set of interneurons and more moderately expressed in a broader population of principal neurons. A representative coronal section through the hippocampal formation of mouse brain is shown. Nonradioactive in situ hybridization was used to identify CB1 mRNA-containing cells. The very highest levels (darkest red) are found in a restricted subset of cells (arrowheads) that are distributed throughout the hippocampal formation. Other studies have shown these to be CCK-containing GABAergic interneurons. Intermediate levels (light pink) are found in the principal neurons of CA1 (arrows) and CA3. Dentate granule cells (asterisks) do not express CB1 mRNA and provide a measure of the background inherent in this technique. Nuclei are identified by toluidine blue counterstaining. This general pattern of expression is recapitulated throughout the neocortex and amygdala. mo, molecular layer; po, polymorphic layer; sg, granule cell layer; slm, stratum lacunosum; so, stratum oriens; sp, stratum pyramidale; sr, stratum radiatum. (Original image provided by Martin Häring.)

The paper by Monory and colleagues in this issue of *PLoS Biology* elegantly addresses this problem using mice with CB1 receptors “knocked out” of specific populations of neurons [21]. These investigators have generated mice whose CB1 gene *(Cnr1)* is flanked by *loxP* sites (“floxed”) to precisely delete CB1 receptors from certain classes of neurons. This control is possible because the DNA sequence between two *loxP* sites will be excised by the DNA recombinase, Cre. By expressing Cre under the control of cell-specific promoters, CB1 receptors can be deleted from restricted populations of cells. In their study, Monory and colleagues crossed these floxed mice with mice carrying Cre recombinase under the control of specific promoters, which are active only in certain neurons. This caused the deletion of CB1 receptors from selected neuronal populations, which was determined by the particular promoter. This group and their collaborators have previously used this approach to demonstrate the importance of CB1 expression in glutamatergic neurons in the neuroprotective effects of endocannabinoids in a seizure model [22] as well as to define the significant role of peripherally expressed CB1 receptors in analgesic responses to cannabinoids [23].

For the present study, in addition to the global knockout of CB1, the researchers used four mouse lines that lacked CB1 in GABAergic neurons, two populations of glutamatergic neurons, or dopamine D1 receptor-expressing neurons. Interestingly, the loss of CB1 from GABAergic neurons, a maneuver that strongly decreases overall CB1 expression and ablates some forms of CB1 receptor-mediated synaptic plasticity [22], had no significant effect on any of the four components of the tetrad. In contrast, the deletion of CB1 from principal glutamatergic neurons strongly attenuated the four components of the tetrad. Within the limitations of this experimental approach (e.g., compensation that may occur due to developmental loss of CB receptors), these results strongly suggest that CB1 expression on glutamatergic neurons is crucial in producing all of the behavioral responses observed in the tetrad, whereas CB1 receptors on GABAergic neurons play little if any role in tetrad responses. Thus, this study, while answering one important question, raises another equally fascinating one: What role (if any) do the high levels of CB1 receptor found on the CCK-positive GABAergic interneurons play in the behavioral responses to THC and cannabis?

What directions might future research using genetically engineered mice take to advance our understanding of the psychoactive effects of cannabis? The most frequently described subjective effects of cannabis intoxication in humans include altered perception (particularly in the passage of time), a sense of relaxation, sociability, and a variety of cognitive effects [24]. A challenge for the field will be to take or develop well-validated mouse behavioral models for these subjective effects and apply them to mice lacking CB1 receptors in specific neuronal populations and treated with THC. Some examples of this would be region-dependent memory tests (particularly those involving the hippocampus as well as extinction of memories), tests of anxiety, and drug discrimination. This line of investigation should help to elucidate the role of these neuronal populations in behaviorally complex actions of cannabis. These studies will powerfully complement those where cannabinoids or their antagonists are microinjected into discrete brain regions to identify the anatomical loci that underlie specific behavioral responses to THC. While experiments similar to those described in the current work are difficult to conduct, including the breeding and characterization of the mouse lines as well as the appropriate performance of behavioral assays, their careful implementation will help define the neural substrate(s) for the psychoactive effects of cannabis that are particularly prominent during human consumption.

## Abbreviations

THC: Δ^9^-tetrahydrocannabinol
